# New insights in addressing cerebral small vessel disease: Associated with extracellular fluid in white matter

**DOI:** 10.3389/fnins.2022.1042824

**Published:** 2022-10-20

**Authors:** Haiyuan Lan, Xinjun Lei, Zhihua Xu, Songkuan Chen, Wanfeng Gong, Yunqi Cai

**Affiliations:** ^1^Department of Radiology, Lishui Hospital of Traditional Chinese Medicine affiliated Zhejiang Chinese Medical University, Lishui, China; ^2^Department of Radiology, Tongde Hospital of Zhejiang Province, Hangzhou, China

**Keywords:** cerebral small vessel disease, diffusion tensor imaging, extracellular fluid, white matter, free water, age

## Abstract

**Objective:**

To explore the role of extracellular fluid, assessed by diffusion tensor imaging (DTI) metrics of free water (FW), in the white matter of patients with cerebral small vessel disease (CSVD).

**Materials and methods:**

The baseline clinical and imaging data of 129 patients with CSVD were collected and reviewed. CSVD MR markers, including periventricular white matter hyperintensity (PWMH), deep white matter hyperintensity (DWMH), cerebral microbleed (CMB), enlarged perivascular space (PVS), and lacunar infarction (LI), were identified, and CSVD burden was calculated. According to total CSVD MR marker score, cases were classified as mild, moderate, or severe. The mean FW and fractional anisotropy (FA) values were calculated using DTI images.

**Results:**

The mean white matter FW was associated with the CSVD MR markers, including PWMH, DWMH, LI and PVS (*P* < 0.05). Moreover, age, hypertension, diabetes mellitus, and FW value were associated with total CSVD MR marker score (*P* < 0.05). Ordinal logistic regression analysis revealed that FW and age were independently associated with CSVD burden (*P* < 0.05). Finally, FW in white matter was associated with FA (r = –0.334, P < 0.001).

**Conclusion:**

Extracellular fluid changes, assessed by DTI metrics of FW in white matter, were associated with CSVD markers and burden. An increased extracellular fluid volume in the white matter was associated with lower FA.

## Introduction

Cerebral small vessel disease (CSVD) is an aging-related disease that affects the small vessels of the brain. It is a leading cause of stroke, cognitive decline, and dementia (Chen et al., 2019). However, the underlying pathophysiology of CSVD remains unclear.

In recent years, alteration of extracellular fluid in white matter, assessed by diffusion tensor imaging (DTI) metrics of free water (FW), has been indicated as a novel imaging marker for Alzheimer’s disease (AD) (Ji et al., 2017; Dumont et al., 2019). Like AD, CSVD is associated with certain changes, such as lower cerebral flood (Lu et al., 2022), increased permeability of blood brain barrier (Thrippleton et al., 2019), and neuroinflammation (Evans et al., 2021). All these factors may lead to increased extracellular fluid volume. Moreover, some studies have indicated that deep medullary vein disruption and venous hypertension are associated with the presence and burden of CSVD (Xu et al., 2020; Ao et al., 2021). Venous hypertension and draining obstruction may lead to interstitial edema and increased extracellular fluid volume. Therefore, we hypothesized that extracellular fluid content is associated with the presence and severity of CSVD.

Furthermore, DTI can identify microstructural changes of white matter *in vivo*. CSVD-associated cognitive decline may be due to demyelination of white matter tracts, destroying the nerve network and ultimately leading to impaired transmission of information within the brain. Microenvironmental changes often cause microstructural changes. In this study, we aimed to explore the relationship between microenvironmental changes in extracellular fluid in white matter and CSVD markers, burden, and microstructural changes in patients with CSVD using multimodal magnetic resonance imaging (MRI).

## Materials and methods

### Patients

The protocol for this study was approved by the Institutional Review Board of our hospital. Each patient or patient proxy provided written informed consent prior to participation in this study. The clinical and imaging data of patients with CSVD gathered from January 2022 to July 2022 were reviewed. The inclusion criteria were as follows: (a) age > 40 years; (b) MR imaging meeting the Standards for Reporting Vascular changes on Neuroimaging (STRIVE) for CSVD (Wardlaw et al., 2013); and (c) presence of at least one vascular risk factor, such as current smoking, diabetes mellitus, hypertension, and hyperlipidemia. The exclusion criteria were as follows: (a) diagnosis of other demyelinating diseases, such as metabolic, hereditary, and inflammatory diseases; (b) severe stenosis or occlusion of an internal carotid artery or large intracranial artery; (c) presence of other brain abnormalities, such as cerebral infarction except lacunar infarction, trauma, tumor, and vascular malformation; and (d) heart, lung, and kidney insufficiency.

### Clinical information

The baseline information of the enrolled patients, including sex, age, and vascular risk factors of hypertension, current smoking, diabetes mellitus, and hyperlipidemia, were collected.

### Magnetic resonance imaging protocol

All patients underwent multimodal MRI, including 3D T1WI, T2WI, T2 Flair, and DTI sequences, on a 1.5 Tesla scanner (MAGNETOM Aera, Syngo Platform VD13A, Siemens Healthcare, Erlangen, Germany) equipped with an eight-channel phased-array head coil. The parameters of the DTI were as follows: repetition time = 3,600 ms, echo time = 95 ms, slice thickness = 3 mm, field of view = 23 cm × 23 cm, matrix = 128 × 128, diffusion directions = 30, b value = 0, 1,000, and 2,000 s/mm^2^.

### Imaging analysis

#### Cerebral small vessel disease MR markers

All images were reviewed separately by two neuroradiologists. Disagreements were resolved by consensus. Using the STRIVE guidelines, we identified white matter hyperintensities (WMH), cerebral microbleed (CMB), perivascular space (PVS), and lacunar infarction (LI). WMH was defined as abnormal hyperintensity of periventricular white matter or deep white matter on T2 FLAIR images. The extent of WMH was assessed and scored using the Fazekas scoring system, and high-grade (H) WMH was defined by a Fazekas score of ≥ 2 in the periventricular white matter (PWMH) and/or ≥ 2 in the deep white matter (DWMH). CMBs were defined as homogeneous hypointensities with an average diameter of 3–5 mm on SWI after excluding calcification, vascular cross section, and abnormal iron deposits. PVS enlargement was defined by the presence of small dot-like or linear fluid signals and small blood vessels on MR images. To evaluate the extent of PVS, a scoring system was used according to the number of PVSs at the level of the maximum number of PVSs in the unilateral vasal ganglia: 0, none-PVS; 1, < 10; 2, < 20; 3, 20–40; 4, > 40. High-grade (H) PVS indicated that the number of enlarged PVSs was > 10. LI was defined as round or ovoid lesions measuring 3–15 mm in diameter, which manifested as hyperintense lesions on T2WI and as hypointense lesions on T1WI.

### Cerebral small vessel disease burden

Cerebral small vessel disease (CSVD) burden was calculated based on the total CSVD MR score, an ordinal scale ranging from 0 to 4, depending on the absence or presence (0 or 1) of each of the four CSVD features (HWMH, CMB, HPVS, and LI). Based on the total CSVD MR score, patients were divided into three groups: mild (total CSVD MR score = 0 or 1), moderate (total CSVD MR score = 2), and severe (total CSVD MR score = 3 or 4). For example, a patient with CMB, HWMH, HPVS, and LI and a total CSVD MR score of 4 was classified into the severe group.

### Extracellular fluid of white matter and fractional anisotropy

First, the DTI images underwent preprocessing steps, including denoising, Gibbs artifact removal, EPI distortion correction, and eddy current correction using MRtrix3.^1^ Thereafter, extracellular fluid was analyzed with the free water elimination two-compartmental model (Hoy et al., 2014), a way to correct partial volume effects of the cerebral spinal fluid and measure the volume of extracellular fluid [free water (FW)] and tissue compartment [fractional anisotropy (FA)] removed the signal of FW, for preprocessed DTI images using the DIPY software.^2^ FW and FA maps were generated. The 3D T1WI images were co-registered as b = 0 (b0) images. Finally, mean white matter FW and FA were calculated with each patient’s white matter mask, including CSVD lesions, segmented by FSL fast^3^ using co-registered 3D T1WI images. The values of FW and FA ranged from 0 to 1. The value of FW reflected the volume of the extracellular fluid. An FW value closer to 1 indicated a markedly increased extracellular fluid volume.

### Statistical analysis

**FIGURE 1:**
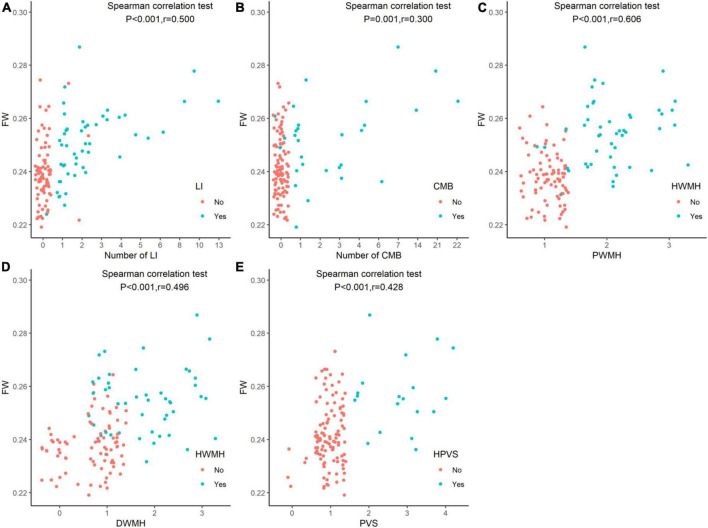
Comparison of extracellular fluid volume of white matter among study participants according to cerebral small vessel disease markers. White matter FW values were associated with the CSVD MR markers of LI **(A)**, CMB **(B)**, PWMH **(C)**, DWMH **(D)**, and PVS **(E)** (*P* < 0.05). FW, free water; LI, lacunar infarction; CMB, cerebral microbleed; DWMH, deep white matter hyperintensity; PWMH, periventricular white matter hyperintensity; HWMH, high-grade white matter hyperintensity; HPVS, high-grade perivascular space.

**FIGURE 2:**
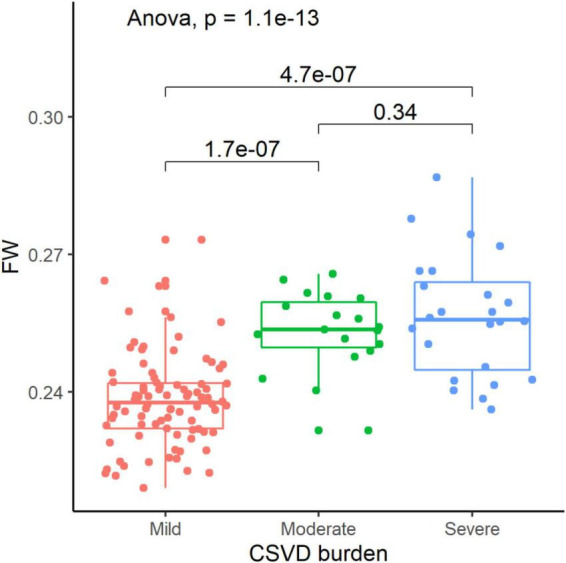
Comparison of extracellular fluid volume of white matter among participants according to cerebral small vessel disease burden. FW, free water.

**FIGURE 3:**
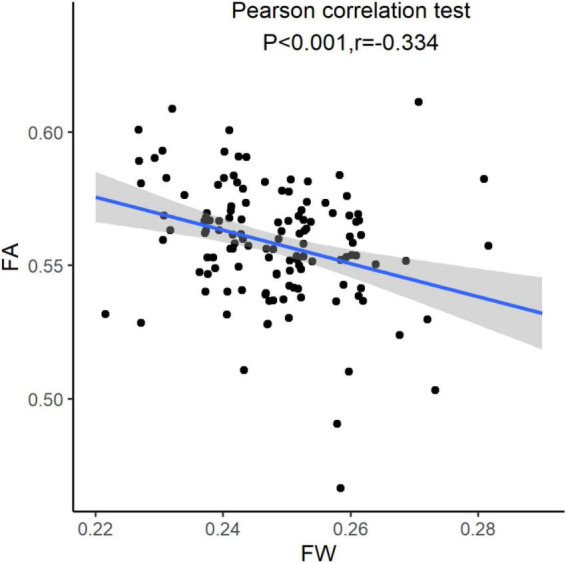
Relationship between extracellular fluid of white matter and fraction anisotropy. FW, free water; FA, fractional anisotropy.

Categorical variables are reported as frequencies and percentages; normally distributed continuous data are reported as means and standard deviations (SD); and non-parametric data are reported as medians and interquartile ranges (IQRs). Associations between FW and CSVD markers were analyzed using Spearman correlation coefficient, and then multivariate linear regression analysis to determine who contribute to FW. associations between FW and total CSVD MR score (mild, moderate, and severe group), age, sex, and vascular risk factors were analyzed using Kruskal-Wallis test or one-way ANOVA test. The relationship between FW and FA was analyzed using the Pearson correlation coefficient. Ordinal regression analysis was performed to identify independent factors associated with total CSVD MR score (mild, moderate, and severe group). A P-value < 0.05 was considered statistically significant. All data analyses were performed using the Statistical Package for Social Sciences for Windows, Version 20 (IBM Corp., Armonk, NY, USA). Figures 1–3 were made using “ggplot2” in R 4.2. To avoid overlapping data points and affect the observation, we also used the “jitter” function when making Figures 1, 2.

## Results

The study enrolled 129 patients, including 60 men. Their mean age was 60 ± 11 years. The number of patients with HWMH, CMB, HPVS, and LI, were 47 (36.43%), 29 (22.48%), 19 (14.72%), and 49 (37.98%) patients, respectively. The median (IQR) total CSVD MR score was 1 (0, 2).

### Extracellular fluid in white matter and cerebral small vessel disease MR markers

White matter FW values were associated with the CSVD MR markers, including PWMH, DWMH, CMB, PVS, and LI (*P* < 0.05, see Figures 1A–E). White matter FW values were higher in patients with HWMH than in patients without HWMH. Similarly, patients with CMB, HPVS, and LI had higher FW values. After multivariate linear regression analysis, PVS, DWMH, PWMH, and number of LI were independently associated with FW (*P* < 0.05, see Table 1); number of CMB was not independently correlated with FW (*P* > 0.05).

**TABLE 1:** Multivariate linear regression analysis for free water in white matter in patients with cerebral small vessel disease.

	β (95% CI)	*P*-value
DWMH	0.158 (0.002, 0.332)	0.046
PWMH	0.341 (0.157, 0.525)	<0.001
Number of LI	0.297 (0.109, 0.486)	0.002
Number of CMB	−0.102 (−0.294, 0.089)	0.292
PVS	0.154 (0.001, 0.309)	0.045

DWMH, deep white matter hyperintensity; PWMH, periventricular white matter hyperintensity; LI, lacunar infarction; CMB, cerebral microbleed; PVS, perivascular space.

### Extracellular fluid in white matter and cerebral small vessel disease burden

The clinical and imaging characteristics and comparisons among study participants according to CSVD burden are shown in Table 2. The number of patients with mild, moderate and severe CSVD were 86 (66.67%), 19 (14.73%), and 24 (18.60%) patients, respectively. The mean FW of mild group (0.24 ± 0.01) significantly differed from moderate (0.25 ± 0.01) and sever (0.26 ± 0.01) group (*P* < 0.05, see Figure 2).

**TABLE 2:** Clinical and imaging features and comparison among participants according to burden of cerebral small vessel disease.

	Burden of cerebral small vessel disease			*P*-value
	Mild (*n* = 86)	Moderate (*n* = 19)	Severe (*n* = 24)	
Male	36 (41.9%)	10 (52.6%)	14 (58.3%)	0.304
Age, years	55.69 ± 10.34	67.95 ± 9.93	68.79 ± 8.65	<0.001
Hypertension, yes	37 (43.0)	16 (84.2)	17 (70.8)	0.001
Diabetes mellitus, yes	12 (14.0)	7 (36.8)	3 (12.5)	0.045
Current smoking, yes	20 (23.3)	3 (15.8)	6 (25.0)	0.739
Hyperlipidemia, yes	22 (25.6)	4 (21.1)	6 (25.0)	0.918
FW	0.24 ± 0.01	0.25 ± 0.01	0.26 ± 0.01	<0.001
FA	0.45 ± 0.02	0.44 ± 0.02	0.43 ± 0.02	0.001

FW, free water; FA, fractional anisotropy.

Age, hypertension, diabetes mellitus, and FW had associations with CSVD burden (*P* < 0.05). After ordinal logistic regression analysis, FW and age were independently associated with CSVD burden (*P* < 0.05) (Table 3).

**TABLE 3:** Multivariate analysis for burden of cerebral small vessel disease.

	β	*P*-value
Age, years	0.861	0.002
Hypertension, yes	−0.526	0.284
Diabetes mellitus, yes	0.151	0.782
FW	1.152	<0.001

FW, free water.

### Extracellular fluid of white matter and fractional anisotropy

Using Pearson correlation coefficient, the extracellular fluid in white matter was associated with FA (*r* = –0.334, *P* < 0.001). A higher volume of extracellular fluid of white matter often had a lower FA (see Figure 3).

## Discussion

CSVD is an aging-related disease. Consistent with findings from other studies, our findings showed that CSVD burden was associated with age. Additionally, changes in extracellular fluid volume, assessed using DTI metrics of white matter FW, was associated with CSVD MR markers (PWMH, DWMH, PVS and LI) and CSVD burden. Moreover, an increased extracellular fluid volume in the white matter was associated with lower FA. Therefore, extracellular fluid volume in the white matter was considered to play an important role in CSVD severity, and increased extracellular fluid was associated with loss of white matter integrity.

Based on the definitions of the CSVD MR markers, the mean white matter FW value can be understood to be higher in patients with higher Fazekas score of PWMH or DWMH. Similarly, patients with higher score of PVS and a greater number of LI had higher mean white matter FW values. Enlarged PVS and LI were filled with fluid, which typically manifested as hyperintense lesions on T2WI and hypointense lesions on T1WI. Although the petrophysical mechanism of WMH remains unclear, a hyperintensity on T2 Flair image, WMH, often indicated an increased water volume, according to the principle of MRI. This finding corroborates similar findings from previous studies (Rau et al., 2021). However, all CSVD MR markers were only lesions in white matter. Several studies (Zhong and Lou, 2016; Khan et al., 2021; Mayer et al., 2022) have shown that microenvironmental and microstructural changes were present in white matter without lesions. Therefore, the global white matter was selected as the region of interest in this study because global white matter extracellular fluid and integrity could reflect pathological changes in CSVD.

CSVD is a clinical syndrome that affects small vessels. It has different clinical presentations and radiomic features (Dey et al., 2019; Wang et al., 2021), which may be due to differences in its pathogenesis and severity. In patients with CSVD, cerebral blood flow is often low (Zhang et al., 2022) and brain tissues are often ischemic, resulting in increased extracellular fluid volume. With respect to the venous system, multiple studies (Chen et al., 2020; Xu et al., 2020; Ao et al., 2021) have shown that deep medullary vein disruption, lumen stenosis, and venous hypertension may lead to interstitial edema and promote CSVD. Moreover, Zhang et al. (2021) indicated that the glymphatic system may play an important role in CSVD. When the glymphatic system was weakened or dysfunctional, cerebrospinal fluid-interstitial fluid exchange and drainage were obstructed and interstitial fluid volume increased. On the other hand, due to blood-brain barrier leakage and glymphatic system dysfunction, brain metabolite (including toxins and αβ proteins) accumulation cannot be effectively cleared in a timely manner, leading to neuroinflammatory reactions and further increase in interstitial fluid volume of the brain tissues (Thrippleton et al., 2019; Zhang et al., 2021). The different pathological mechanisms underlying the development of CSVD result in increased extracellular fluid volume. Therefore, change in cerebral extracellular fluid volume is probably a comprehensive response to CSVD.

Moreover, the increased extracellular fluid volume in the white matter was associated with lower FA. Previously, several studies (Liu et al., 2019, 2020; Kerkhofs et al., 2021) found that microstructural changes were associated with cognitive impairment in patients with CSVD. Therefore, we sought to understand the association between the risk factors and microstructural changes. According to our study, the increased extracellular fluid volume could cause an accumulation of harmful substances, such as plasma proteins, which are toxic to surrounding white matter microstructures, including myelin and axons. Increased extracellular fluid volume possibly occurs first, followed by demyelination and axonal damage within the white matter. Therefore, microstructural changes may be mediated by accumulation of extracellular fluid in patients with CSVD. To corroborate these findings, further studies are needed.

This study had some limitations. First, the sample size was relatively small and did not include patients with CSVD and associated cognitive decline or dementia. Second, participants were not followed up to study the relationship between CSVD progression and changes in extracellular fluid volume and FA in the white matter. Although FW in global white matter may provide more potential pathological changes to reflect CSVD burden, FW and FA in normal appearing white matter may be more informative to explore CSVD progression. Further multi-center studies with large sample sizes are needed to address these limitations.

## Conclusion

Changes in cerebral extracellular fluid volume, assessed using DTI metrics of white matter FW, may be a comprehensive response to CSVD. Additionally, an increased extracellular fluid volume was associated with lower FA.

## Data availability statement

The original contributions presented in this study are included in the article/supplementary material, further inquiries can be directed to the corresponding author.

## Ethics statement

The studies involving human participants were reviewed and approved by Tongde Hospital of Zhejiang Province. The patients/participants provided their written informed consent to participate in this study.

## Author contributions

HL, XL, and ZX conceived the project idea and wrote the manuscript. ZX provided the critical suggestions for the design of the experiments. HL, XL, WG, SC, and YC collected the imaging and clinical data. HL, XL, ZX, and SC provided the imaging analysis. XL and ZX supervised the project. All authors contributed to the article and approved the submitted version.

## Footnotes

1
https://www.mrtrix.org/

2
https://dipy.org/

3
https://fsl.fmrib.ox.ac.uk/fsl/fslwiki/FAST
